# Whole-Exome Sequencing of Pakistani Consanguineous Families Identified Pathogenic Variants in Genes of Intellectual Disability

**DOI:** 10.3390/genes14010048

**Published:** 2022-12-23

**Authors:** Maria Asif, Maryam Anayat, Faiza Tariq, Tanzeela Noureen, Ghulam Naseer Ud Din, Christian Becker, Kerstin Becker, Holger Thiele, Ehtisham ul Haq Makhdoom, Pakeeza Arzoo Shaiq, Shahid M. Baig, Peter Nürnberg, Muhammad Sajid Hussain, Ghazala Kaukab Raja, Uzma Abdullah

**Affiliations:** 1 Cologne Center for Genomics (CCG), University of Cologne, Faculty of Medicine and University Hospital Cologne, 50931 Cologne, Germany; 2 Center for Molecular Medicine Cologne (CMMC), University of Cologne, Faculty of Medicine and University Hospital Cologne, 50931 Cologne, Germany; 3 University Institute of Biochemistry and Biotechnology (UIBB), PMAS-Arid Agriculture University Rawalpindi (PMAS-AAUR), Rawalpindi 46300, Pakistan; 4 Neurochemical Biology and Genetics Laboratory (NGL), Department of Physiology, Faculty of Life Sciences, Government College University, Faisalabad 38000, Pakistan; 5 Human Molecular Genetics Laboratory, Health Biotechnology Division, National Institute for Biotechnology and Genetic Engineering (NIBGE) College, PIEAS, Faisalabad 38000, Pakistan; 6 Department of Biological and Biomedical Sciences, The Aga Khan University, Karachi 74800, Pakistan; 7 Pakistan Science Foundation (PSF), Islamabad 44050, Pakistan

**Keywords:** intellectual disability, whole-exome sequencing, consanguineous, *MBOAT7*, *TRAPPC9*

## Abstract

Intellectual disability (ID) is a condition of significant limitation of cognitive functioning and adaptive behavior, with 50% of etiology attributed to genetic predisposition. We recruited two consanguineous Pakistani families manifesting severe ID and developmental delay. The probands were subjected to whole exome sequencing (WES) and variants were further prioritized based on population frequency, predicted pathogenicity and functional relevance. The WES data analysis identified homozygous pathogenic variants in genes *MBOAT7* and *TRAPPC9*. The pathogenicity of the variants was supported by co-segregation analysis and in silico tool. The findings of this study expand mutation spectrum and provide additional evidence to the role of MBOAT7 and TRAPPC9 in causation of ID.

## 1. Introduction

Intellectual disability (ID) is a condition of significant limitation of cognitive functioning and adaptive behavior (intelligent quotient lower than 70) manifesting before the age of 18 years old [1]. ID has a very heterogenous clinical presentation (syndromic and non-syndromic) and its etiology is attributed to both environmental and inherited aberrations [2]. The global incidence of ID ranges from 1 to 3% [3]. Patients need lifetime care that, in addition to the psychological toll it takes, casts serious financial strain on the families and healthcare system alike [4]. ID is observed in all patterns of mendelian inheritance and with a genetic etiology as diverse as large chromosomal abnormalities, submicroscopic copy number variants, and monogenic forms due to pathogenic variants in single genes [5,6,7]. The autosomal recessive form of ID (ARID) has been rare and therefore the least explored [8]. With the increasing feasibility of next-generation sequencing, more cases of ARID are being explored, which is revealing its ever increasing genetic heterogeneity [8]. According to SysNDD (https://sysndd.dbmr.unibe.ch/Entities (accessed on 30 October 2022)), 995 genes have been definitely linked to ARID, though the magnitude of candidate genes greatly exceeds this number [9] and is expected to be around 3000 [8]. This is reflected in a consistently low diagnostic yield of genetic diagnostic for ID, which has not exceeded 50%, at best [10,11]. In addition, an increasing adaption of next-generation sequencing for prenatal diagnosis and stronger evidence for pathogenicity of reported variants are required for accurate risk estimate; it urges the need for investigation of more informative pedigrees and deep phenotyping.

Here, we report two consanguineous families with severe intellectual disability that were analyzed with next-generation sequencing, segregating a novel and known mutation in the genes of ARID.

## 2. Materials and Methods

### 2.1. Subjects

The study was approved by “The Ethics Committee for the Use of Human Subjects” of Pir Mehr Ali Shah Arid Agriculture University Rawalpindi (PMAS-AAUR). We recruited two consanguineous families from the remote area of Punjab province of Pakistan. The families were educated about the aim and nature of the study in their native language and written informed consent was obtained as per the rules described in the Declaration of Helsinki. From all consenting individuals, peripheral blood was collected in EDTA-coated tubes and stored at 4 °C.

### 2.2. Whole-Exome Sequencing

We extracted genomic DNA from blood samples of all individuals using phenol chloroform extraction method. The DNA of affected individual IV-1 from family A and IV-4 from family B were subjected to whole-exome sequencing. For this purpose, we used Agilent SureSelectXT HS Human All Exon V8 enrichment kit and the samples were subsequently run on an Illumina HiSeq 2000 sequencing system (paired-end reads, 2 × 100 bp) and data were analyzed as described before [12]. For variant interpretation, we used VARBANK, our in-house database and analysis platform (https://varbank.ccg.uni-koeln.de/varbank2/ (accessed on 1 July 2022)) of the Cologne Center of Genomics (CCG), University of Cologne, Germany.

### 2.3. Co-Segregation Analysis 

The candidate variants were further analyzed through bidirectional Sanger sequencing of variant-spanning genomic fragments in all family members. The reference sequences were retrieved from the UCSC Human genome browser and sequences were analyzed through UGENE (Version 45.0). 

### 2.4. In Silico Analysis

To assess the likely pathogenicity of the variants, multiple in silico tools were employed, using web interface of Variant effect predictor (VEP) available at Ensemble (https://asia.ensembl.org/info/docs/tools/vep/index.html (accessed on 15 August 2022)) and Varsom (https://varsome.com/ (accessed on 14 October 2022)).

## 3. Results

### 3.1. Clinical Findings 

**Figure 1:**
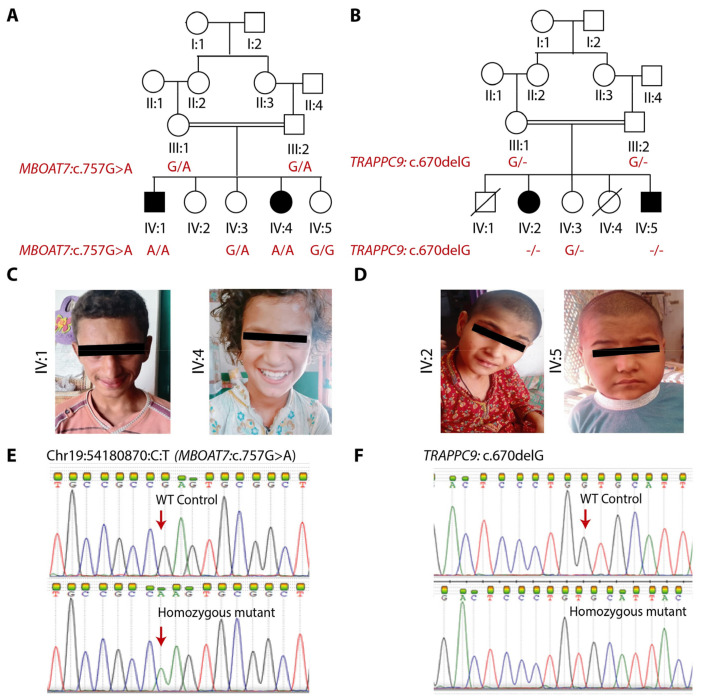
Clinical and genetic findings of families A and B (**A**) Pedigree of family A along with genotypes of candidate variant in *MBOAT7* showing its co-segregation. (**B**) Pedigree of family B along with genotypes of candidate variant in *TRAPPC9* (**C**) Frontal poses of patients of family A showing normal facial features. (**D**) Frontal poses of affected individuals of family B without any signs of facial deformities. (**E**) Sanger traces of the genomic region of *MBOAT7* showing identified variant in the affected member compared to the control. Arrow shows the position of the mutant nucleotide. (**F**) Sanger traces for mutation spanning region in of *TRAPPC9* showing wild-type sequence in a control individual in the upper panel, whereas it shows homozygous deletion c.670delG in the lower panel, as observed in both patients.

The families, here designated as A and B (pedigrees provided in Figure 1A,B), consisted of two affected individuals each, born as a result of full-term, uneventful pregnancies to consanguineous couples. All four patients presented global developmental delay, severe intellectual disability and speech impairment. The facial features of the patients were evaluated by a clinical geneticist and confirmed to be normal, as shown in Figure 1C,D. The patients also presented variable features, such as microcephaly, reduced height, walking abnormality and seizures. The clinical features of each individual are detailed in Table 1.

### 3.2. Genetic Findings 

WES analysis identified a presumable homozygous loss-of-function variant *MBOAT7*, NM_024298.4:c.757G>A (Figure 1E). It is a missense variant and is predicted to substitute a highly conserved amino acid residue (p.(Glu253Lys)). Sanger sequences confirmed the homozygosity of the variant in both patients, whereas parents were heterozygous carrier (Figure 1A,E). For family B, a homozygous variant in another gene, *TRAPPC9*, NM_001160372.3,c.670delG, was identified. It is predicted to cause frameshift followed by premature termination p.(Val224Cysfs*13). The Sanger sequencing (Figure 1F) showed co-segregation of the variant, confirming the autosomal recessive mode of inheritance, as shown in Figure 1B.

### 3.3. In Silico Findings 

Both of the candidate variants showed strong support for predicted pathogenicity from tools. The findings of in silico tools for the variant MOBAT7, NM_024298.5: c.757G>A, p.(Glu253Lys) are provided in Table 2. The variant is highly conserved across the animal kingdom (the conservation in some representative species is shown in Supplementary Figure S1). The *TRAPPC9*, NM_001160372.3, c.670delG is predicted to be likely pathogenic as per ACMG classification (9 points = 9 P − 0 B) and its conservation score on phyloP100 is 5.888. 

## 4. Discussion

The study reports the clinical and genotypic findings of two consanguineous families with representative symptoms of intellectual disability. Although ID is a well-characterized disorder, the genetic and clinical heterogeneity of ID in general and its autosomal recessive form are partially documented [8]. The human brain receives the majority of genomic expression and many of these genes are underexplored. The genomic dissection of ARID holds the potential to generate a wealth of data that can contribute to a better understanding of the development and function of the human brain [8]. With the evolving application and down pricing of next-generation sequencing, it is increasingly possible to analyze smaller pedigrees, thus increasing the possibility of novel findings.

Family A segregated missense mutation in *MBOAT7*. The gene encodes membrane-bound O-acyltransferase domain-containing 7 (MBOAT7) protein, which is lysophospholipid acyltransferase 7 enzyme, functions for the incorporation of arachidonic acid into glycophosphatidylinositol and is involved in phosphatidylinositol acyl-chain remodeling in the Lands cycle [13]. The process is highly regulated: deficiency of MBOAT7 leads to elevated levels of arachidonic acid that compromise cellular physiology [14], including that of neurons. In 2016, biallelic loss-of-function mutations in this gene were identified to cause ARID by Johansen et al. [15], and since then, 19 different mutations have been identified in 51 patients, causing overlapping symptoms of developmental delay, intellectual disability, seizures and with variable reports of structural brain anomalies assessed by magnetic resonance imaging (MRI). As detailed in Supplementary Table S1, the patients with biallelic mutations in *MBOAT7* also present the most frequent co-segregation of seizures (40/51), speech impairment (45/51), delayed acquisition of motor milestones (23/51), occasionally autistic features (16/51), and microcephaly (01/51). The mutation observed in family A, *MOBAT7*: NM_024298.5: c.757G>A; p.(Glu253Lys), has been previously reported in an unrelated Pakistani family [16]. The patients of the reported family manifested generalized tonic colonic seizures with the latest age of onset being 5 years, severe global developmental delay, severe intellectual disability and mild atrophy of the cerebellar cortex [16]. The patients of family A presented symptoms overlapping with the aforementioned reported patients, including severe intellectual disability, developmental delay, and speech impairment/deficiency. The patients IV:1, however, never developed epilepsy, even at the age of 16 years. The patients also had a normal walk and the disease appears to be static. 

MBOAT family proteins, including MBOAT7, adopt a conserved helical core structure, comprising an extracellular funnel and intracellular tunnel [17]. Multiple in silico tools for the modeling of the mutation indicated that the substitution of p.(Glu253Lys) may cause the mutant protein to form ectopic hydrogen bonds that restrict its conformational flexibility of funnel, ultimately compromising its function [16], leading to the observed phenotype. 

Family B shows a novel homozygous mutation in *TRAPPC9*. Its encoded protein is a subunit of the TRAPP complex (TRAPPC), a guanine nucleotide exchange factor for rab proteins that operate in secretory, endocytic, and autophagic pathways [18]. TRAPPC9 is the main component of TRAPP complex type II, also knows as TRAPII, which facilitates endoplasmic reticulum (ER)-to-Golgi vesicular transport, the transport within the Golgi, and the transport between Golgi and endosomes [19]. Biallelic loss-of-function mutations have been observed with intellectual disability, along with variable clinical co-segregation of obesity, hypoplastic corpus callosum, microcephaly, behavioral changes, and dysmorphic features [19,20,21]. The clinical features of the reported families have been summarized in Supplementary Table 2. As apparent in the table, the most frequent co-segregating features include microcephaly (39/44), delay or lack of speech acquisition (33/44), and delay of motor milestone or walking abnormality (12/44), whereas less frequently observed features included abnormalities of white matter or thinning of corpus callosum (18/44), more rarely autistic features (4/44) and seizures (2/44). Trappc9-deficient mice have also been observed to have behavioral deficits such as global delay of intellectual development, postnatal microcephaly, thin corpus callosum with imbalance in dopamine D1 and D2 receptor containing neurons [22]. *Trappc9*-null mice also showed decreased contents of neural stem cells in the subventricular zone of the lateral ventricle and the sub granular zone of the dentate gyrus [23], which likely explains the structural abnormalities of the brain observed in patients too.

The longest isoform of wild-type TRAPPC9 consists of 1148 amino acid residues. The mutation identified in family B is NM_001160372.3:c.670delG, which is predicted to shift the wild-type frame that will run into premature stop codon, making a polypeptide chain of 337 amino acids only p.(Val224Cysfs*13). Due to such an early termination, the mutant transcript is likely to be subjected to non-sense mediated decay. Thus, it will function as a null mutation, causing the complete absence of the protein.

Various types of homozygous null mutations of *TRAPPC9* that lead to ARID along with microcephaly and occasionally seizure (Supplementary Table S2) have been reported. Our patients exhibited similar symptoms of microcephaly, short stature, seizures and intellectual disability.

In conclusion, the study adds to the phenotypic and genetic spectrum of *MBOAT7*- and *TRAPPC9*-related intellectual disability. This knowledge adds evidence to the role of these two genes in the nervous system’s development. It also adds to the genetic spectrum of ID in the Pakistani population.

## Figures and Tables

**Table 1:** Clinical information of the families A and B.

Family IDs	Family A		Family B	
Mutation	*MBOAT7* (NM_024298.4: c.757G>A; p.(Glu253Lys))		*TRAPPC9* (NM_001160372.3: c.670delG; p.(Val224Cysfs*13)	
Patient ID	IV:1	IV:4	IV:2	IV:5
Sex	Male	Female	Female	Male
Age at examination	16 years	10 years	10 years	4 years
Height	175.25 cm	137.20 cm (−0.3 SD)	121.90 cm (−3 SD)	86.35 cm (−4 SD)
HC	50.10 cm (−4 SD)	53.35 cm (+1 SD)	45.72 cm (−5 SD)	43.20 cm (−7 SD)
Intellectual disability	Severe	Severe	Severe	Severe
Self-care	No	No	No	No
Self-feed	No	No	No	No
Gestational history	Unremarkable	Unremarkable	Unremarkable	Unremarkable
Sitting	1 year	2 years	2 years	2 years
Walking	3 years	3.5 years	5 years	Not attained
Speech Impairment	Yes	Yes	Yes	Yes
Visual Impairment	No	No	No	No
Ambulation Difficulty	No	No	No	No
Facial Dysmorphism	No	No	No	No
Aggressive	Yes	Yes	N/A	N/A
Seizures	No	Yes, started at the age of 5 months	Yes, onset at the age of 4 years	Yes, onset at the age of 1.5 years
Skin anomalies	No	No	No	No

**Table 2:** In silico analyses for pathogenicity of *MBOAT7*, NM_024298.5: c.757G>A, p.(Glu253Lys).

Prediction Tool	Prediction and Score
gnomAD frequency	1/152, 184 (0.000006571)
CADD	27.1
PolyPhen-2	Probably damaging (0.999)
DANN	Pathogenic (0.9993)
EIGEN	Pathogenic (0.7627)
SIFT	Deleterious
Mutation Taster	Disease-causing (56)
Polyphen-2	Probably damaging (1.000)
Conservation Scores	phyloP100: 4.065
EIGEN PC	Pathogenic (0.7029)
PROVEAN	Uncertain (3.28, 3.46, 3.05, 3.03)
ACMG Classification	Uncertain significance (PM1, PP3 and BP1)
SNAP^2^	Neutral −41 (72%)
DEOGEN2	Uncertain (Damaging, Tolerated) 0.5828, 0.4976
MetaRNN	Pathogenic
FATHMM-MKL	Pathogenic (0.9838)
FATHMM-XF	Uncertain (0.8913)
MutPred	Pathogenic (0.84)
PANTHER	(Probably damaging) (0.74)
REVEL	Pathogenic (0.7459)
BayesDel addAF	Pathogenic (addAF score 0.2137)

## Data Availability

Not applicable.

## Supplementary Materials

The following supporting information can be downloaded at: https://www.mdpi.com/article/10.3390/genes14010048/s1, Figure S1: Conservation of MBOAT7 amino acid residue p.Glu253 (shown in red) across different orthologs; Table S1: Clinical and mutational spectrum of *MBOAT7*; Table S2: Clinical and mutational spectrum of *TRPPC9*.
