# Supplementary figures and images for: Whole-Exome Sequencing of Pakistani Consanguineous Families Identified Pathogenic Variants in Genes of Intellectual Disability
Source: Genes (Basel). 2022 Dec 23;14(1):48. doi: 10.3390/genes14010048 (PMC9858807; doi:10.3390/genes14010048)

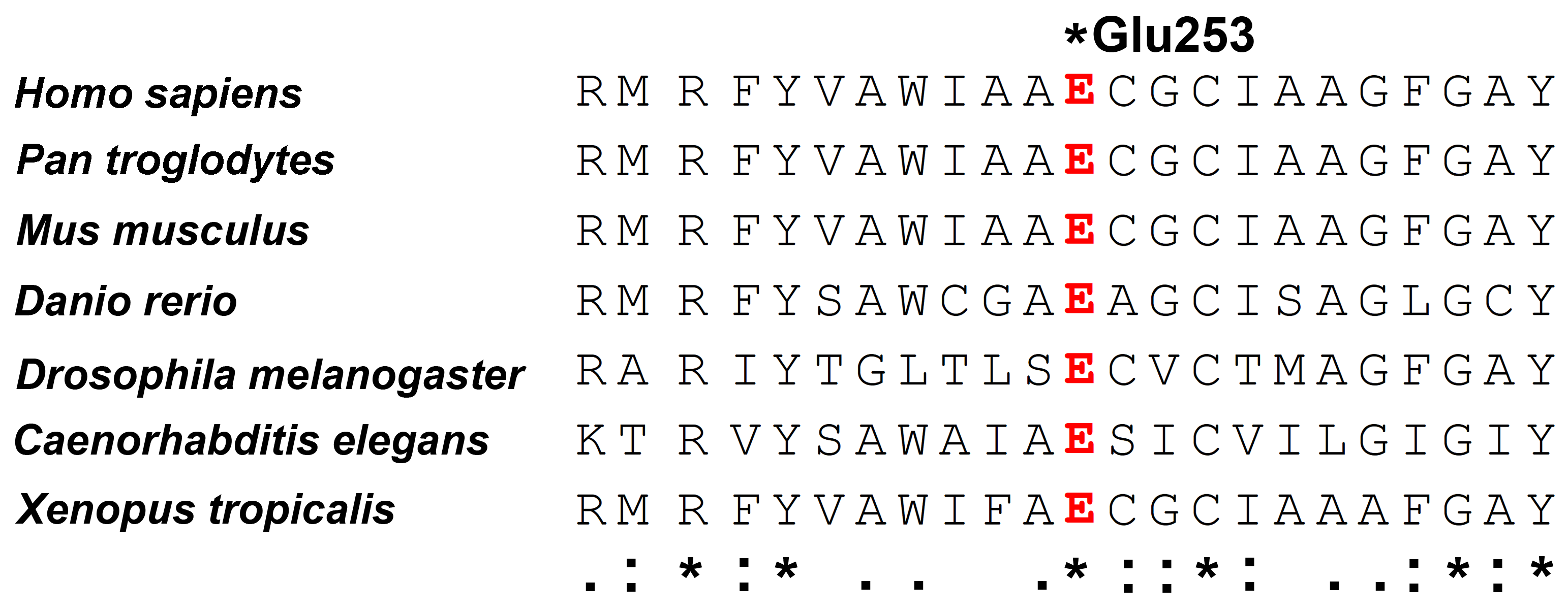

Supplement: Supplementary file 1 [file genes-14-00048-s001.zip › genes-2033021-supplementary/Supplementary files/Figure S1 Conservation of MBOAT7 amino acid residue p.Glu253 (shown in red) across different orthologs.tif]
